# Supplementary material for: Comparative genomics provides new insights into the diversity, physiology, and sexuality of the only industrially exploited tremellomycete: Phaffia rhodozyma
Source: BMC Genomics. 2016 Nov 9;17:901. doi: 10.1186/s12864-016-3244-7 (PMC5103461; doi:10.1186/s12864-016-3244-7)
Supplement: Additional file 6: — List of orphan genes with links to PFAM (related to Additional file 1: Table S1). (ZIP 1428 kb) [file 12864_2016_3244_MOESM6_ESM.zip › BLAST_HTML_FTR/G02353_P.html]

BLAST Search Results


```
BLASTP 2.2.27+


Reference:
Stephen F. Altschul, Thomas L. Madden, Alejandro A. Schäffer,
Jinghui Zhang, Zheng Zhang, Webb Miller, and David J. Lipman (1997),
"Gapped BLAST and PSI-BLAST: a new generation of protein database
search programs", Nucleic Acids Res. 25:3389-3402.


Reference for
composition-based statistics:
Alejandro A. Schäffer, L. Aravind, Thomas L. Madden, Sergei
Shavirin, John L. Spouge, Yuri I. Wolf, Eugene V. Koonin, and
Stephen F. Altschul (2001), "Improving the accuracy of PSI-BLAST
protein database searches with composition-based statistics and
other refinements", Nucleic Acids Res. 29:2994-3005.


Database: nr
           71,551,133 sequences; 26,053,659,533 total letters


Query= G02353_P

Length=282
                                                                      Score     E
Sequences producing significant alignments:                          (Bits)  Value

ref|XP_007002690.1|  hypothetical protein TREMEDRAFT_60031 [Treme...  40.4    1.8  
gb|EHA24280.1|  hypothetical protein ASPNIDRAFT_39673 [Aspergillu...  39.3    4.4  
ref|WP_030079212.1|  hypothetical protein [Streptomyces baarnensis]   39.3    4.4  


 >ref|XP_007002690.1| hypothetical protein TREMEDRAFT_60031 [Tremella mesenterica DSM 
1558]
 gb|EIW71090.1| hypothetical protein TREMEDRAFT_60031 [Tremella mesenterica DSM 
1558]
Length=715

 Score = 40.4 bits (93),  Expect = 1.8, Method: Compositional matrix adjust.
 Identities = 33/130 (25%), Positives = 54/130 (42%), Gaps = 31/130 (24%)

Query  166  IPTLLPVNETTTEGEEINIEGDGPFEILGLKVPKRPIKPGDE-----------------A  208
            +P + P+  T  +    +  G+G   + G+ +P +P+ PG E                 A
Sbjct  340  VPAMKPLKLTVEKETVASTSGEGEIWVQGIMIPPKPVPPGAEECCMNSCVNCVYNLYADA  399

Query  209  LEAVRVALLRAT-------VPRTAWPVRVIELD-----EETSQSGGKPDPFAGLDPGLRA  256
            LEA   AL  A        +PR  WP  V+ ++     E  ++   K +   GLDP + A
Sbjct  400  LEAYTAALSEAVKALKIADIPRKEWPAAVLAVERKGPGEMVAEEERKME--EGLDPSMAA  457

Query  257  FKEFEAKLKR  266
            F   E  L++
Sbjct  458  FLALEGMLRK  467


>gb|EHA24280.1| hypothetical protein ASPNIDRAFT_39673 [Aspergillus niger ATCC 
1015]
Length=1340

 Score = 39.3 bits (90),  Expect = 4.4, Method: Compositional matrix adjust.
 Identities = 35/113 (31%), Positives = 52/113 (46%), Gaps = 5/113 (4%)

Query  92   TSSVSGLTSSALPSSISSLSTSPLSGSLPNIAFPSTSSTSSSSSSSSSILPPSTTPPTPL  151
            TSS      +A   +   LST   +GSL N   P T+S    +SS+S + PPS +   P+
Sbjct  669  TSSQDRQNGAAAHKNSDILSTQVSNGSLHNDTTPYTTSDMGFNSSASGVQPPSVSINGPM  728

Query  152  HTSLPADPIEIQARIPTLL--PVNETTTEGEEINIEGDGPFEILGLKVPKRPI  202
            H S  +DP      +P+ +   + E    G E+    D P E   +  PKR +
Sbjct  729  HNSQKSDPFSSYREMPSSISSKIGEVVVAGIEMR---DTPAEQPTMPRPKRRV  778


>ref|WP_030079212.1| hypothetical protein [Streptomyces baarnensis]
Length=447

 Score = 39.3 bits (90),  Expect = 4.4, Method: Compositional matrix adjust.
 Identities = 56/201 (28%), Positives = 80/201 (40%), Gaps = 25/201 (12%)

Query  66   SRDESASHDERQKVMPSVSTTTSPIRTSSVSGLTSSALPSSISSLSTSPLSGSLPNIAFP  125
            +R +  S  E  K  PS S T    +    SG   +A P   +S S S       + A P
Sbjct  82   TRSDEPSESESPKATPSPSATAKDDKKDGASGKDDTATPKPSASGSASDADEDTADGAKP  141

Query  126  STSSTSSSSSSSSSILPPSTTPPTPLHTSLPADPIEIQARIPTLLPVNETTTE--GEEIN  183
             T   S+ ++       PS T PTP  +  P DP+ +   +  L    + T +  G+E  
Sbjct  142  GTEDKSAQAAQEKPAETPSAT-PTPTRSKNPLDPLGLGDALKDLFDGPDKTDDEAGQEAG  200

Query  184  IEG-DGPFEILGLKVPKRPIKPGDEALEAVRVALLRA--TVPRTAWPVR---------VI  231
             E    P      + PK   +P DEA + V+ A  +A  T  +TA  +R         V 
Sbjct  201  DEKPASPSPSPTTEAPKASDEPEDEAKDPVKDAADQAKETSDKTADAIREAAGKAGKDVE  260

Query  232  ELDEETSQSGGKPDPFAGLDP  252
            ELDE+            GLDP
Sbjct  261  ELDEDVK----------GLDP  271


Lambda      K        H        a         alpha
   0.313    0.131    0.379    0.792     4.96 

Gapped
Lambda      K        H        a         alpha    sigma
   0.267   0.0410    0.140     1.90     42.6     43.6 

Effective search space used: 2072188307766


  Database: nr
    Posted date:  Sep 23, 2015 12:05 AM
  Number of letters in database: 26,053,659,533
  Number of sequences in database:  71,551,133


Matrix: BLOSUM62
Gap Penalties: Existence: 11, Extension: 1
Neighboring words threshold: 11
Window for multiple hits: 40
```
